# Detached epithelial cell plugs from the upper respiratory tract favour distal lung injury in Golden Syrian hamsters (*Mesocricetus auratus*) when experimentally infected with the A.2 Brazilian SARS-CoV-2 strain

**DOI:** 10.1590/0074-02760240100

**Published:** 2024-10-21

**Authors:** Marcelo Pelajo-Machado, Alexandre dos Santos da Silva, Daniela del Rosario Flores Rodrigues, Milla Bezerra Paiva, Rodrigo Muller, Luciana Jesus da Costa, Pedro Paulo Abreu Manso, João Paulo Rodrigues dos Santos, Emanuelle de Souza Ramalho Ferreira da Silva, Arthur Daniel Rocha Alves, Jaqueline Mendes Oliveira, Marcelo Alves Pinto

**Affiliations:** 1 Fundação Oswaldo Cruz-Fiocruz, Instituto Oswaldo Cruz, Laboratório de Medicina Experimental e Saúde, Rio de Janeiro, RJ, Brasil; 2 Fundação Oswaldo Cruz-Fiocruz, Instituto Oswaldo Cruz, Laboratório de Desenvolvimento Tecnológico em Virologia, Rio de Janeiro, RJ, Brasil; 3 Fundação Oswaldo Cruz-Fiocruz, Bio-Manguinhos, Vice-Diretoria de Inovação, Departamento Experimental e Pré-Clínico, Laboratório de Ensaios Pré-Clínicos, Rio de Janeiro, RJ, Brasil; 4 Universidade Federal do Rio de Janeiro, Instituto de Microbiologia Paulo de Góes, Departamento de Virologia, Laboratório de Genética e Imunologia das Infecções Virais, Rio de Janeiro, RJ, Brasil; 5 Fundação Oswaldo Cruz-Fiocruz, Central Analítica COVID-19, Rio de Janeiro, RJ, Brasil

**Keywords:** A2 SARS-CoV-2 Brazilian strain, rodent model, Mesocricetus auratus, pathogeny, infectious epithelial plugs, segmentary pneumonia, acute lung injury

## Abstract

**BACKGROUND:**

The Golden Syrian hamster (*Mesocricetus auratus*), Ferrets (*Mustela putorius furo*), and macaques have been described as useful laboratory animals naturally susceptible to severe acute respiratory syndrome coronavirus 2 (SARS-CoV-2) infection.

**OBJECTIVES:**

To study the mechanism of lung injury, we describe the histopathological features of SARS-CoV-2 infection in Golden Syrian hamsters inoculated intranasally with the A.2 Brazilian strain.

**METHODS:**

Hamsters were intranasally inoculated with the A.2 variant and euthanised at 3-, 5-, 10- and 15-days post-inoculation. The physical examination and body weight were recorded daily. Neutralising antibodies and viral RNA load of the respiratory tract were assessed during necropsies.

**FINDINGS:**

The coronavirus disease 2019 (COVID-19) model presented body weight loss, high levels of respiratory viral RNA load, severe segmentary pneumonitis, and bronchial fistula besides lymphatic trapping and infiltration, like the human SARS-COV-2 pathogenesis. The presence of subepithelial lymphoeosinophilic infiltrate was highlighted in our results; it contributed to the detachment of SARS-CoV-2 nucleocapsid-positive epithelial cells resulting in the infectious cell plugs.

**MAIN CONCLUSIONS:**

The SARS-CoV-2 caused segmentary pneumonia and vascular damage. In our comprehension, the infectious cell plugs, as being aspirated from the upper respiratory tract into the terminal bronchial lumen, work as a “Trojan horse”, thus contributing to the dissemination of the SARS-CoV-2 infection into specific regions of the deep lung parenchyma.

Severe acute respiratory syndrome coronavirus 2 (SARS-CoV-2) infection can induce severe endothelial injury and vasculitis in the lung parenchyma, with the presence of intracellular virus and disrupted cell membranes, followed by microvascular and macrovascular thrombosis, and intussusceptive angiogenesis.^1^ Therefore, those changes in the intrapulmonary vascular network represent a mechanism of coronavirus disease 2019 (COVID-19) hypoxia.^2^ Rhesus monkeys,^3^ K18-human angiotensin-converting enzyme 2 (hACE2) mice^4^ and old Golden Syrian hamsters (*Mesocricetus auratus*) challenged with SARS-CoV-2^5^ and ferrets^6^ have shown a similar picture, with the lungs as the main target of the infection. Moreover, experimentally, and naturally infected aged hamsters and Ferrets have reproduced the progression of human lung disease.^6^ ^,^ ^7^ ^,^ ^8^ ^,^ ^9^

Here, Golden Syrian hamsters were experimentally infected with A.2 SARS-CoV-2 Brazilian strain. The A.2 strain emerged in Brazil during the first wave of the COVID-19 epidemic in 2020, the phylogeographic analysis also pointed out that the A.2 Brazilian strain was most probably introduced from Spain.^10^ ^,^ ^11^ The histopathological features of SARS-CoV-2 infection in Golden Syrian hamsters indicated the upper epithelial plugs contributed to the deep lung dissemination of the SARS-CoV-2, forming segmentary pneumonia induced by A.2 variants that isolated in Rio de Janeiro during the epidemic of COVID-19 in Brazil.

## MATERIALS AND METHODS

*Inocula and experimental design* - A.2 Brazilian SARS-CoV-2 strain (GISAID: EPI_ISL_528539/2020-03-19) were prepared from a nasopharyngeal swab clinical specimen of a patient presenting mild COVID-19 symptoms. Viral stock was generated after viral passages and titrated by plaque-forming assay in Vero-hAce-2/human transmembrane serine protease 2 (hTMPRSS-2) cells. Adult male and female Golden Syrian hamsters (older than 1.8 years) were kept in cages with three animals in climate-controlled rooms (temperature of 21 ± 3ºC and humidity 55 ± 15%) with a 12 h light/dark cycle. Animals were fed with a commercial hamster diet and water was provided *ad libitum*. Clinical samples and viral stocks were manipulated in a biosafety level 3 (BSL-3) facility of Fiocruz-IOC and the Golden Syrian hamsters were housed in the animal biosafety level 3 (ABSL-3) facility of Laboratório de Ensaios Pré-Clínicos (Bio-Manguinhos/Fiocruz).

**Fig. 1:**
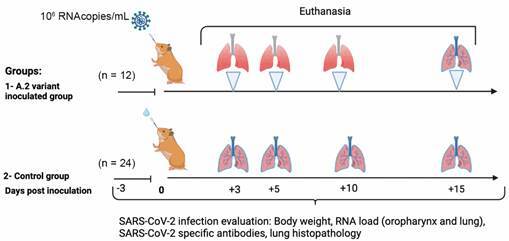
experimental design of A.2 Brazilian severe acute respiratory syndrome coronavirus 2 (SARS-CoV-2) strain infection in Golden Syrian hamster.

The experimental design was realised with two groups (infected and negative control) of Golden Syrian hamsters. The infected group were inoculated intranasally with 50 µL of viral suspensions [1,5 x 10^6^ plaque forming units (PFU)/mL] of the A.2 Brazilian SARS-CoV-2 strain and the negative control group was inoculated intranasally with 50 µL of phosphate-buffered saline (PBS). The animals were euthanised at 3-, 5-, 10- and 15-days post-inoculation (DPI). For euthanasia, all animals were anaesthetised with ketamine hydrochloride at 50 mg/kg (Cetamin, Syntec, São Paulo, Brazil) and xylazine hydrochloride at 5 mg/kg (Xilasin, Syntec, São Paulo, Brazil). After the anesthesia, the hamsters were euthanised under deep barbiturate anaesthesia with sodium thiopental 2.5% at 150 mg/kg (Thiopentax, Cristália, São Paulo, Brazil) which was delivered intraperitoneally. Subsequently, cardiac punctures were performed, and the animals were euthanised by total exsanguination. The chronogram of the study is depicted in Fig. 1.

*Clinical and virological monitoring* - After inoculation, the physical examination, clinical manifestations, and body weight were measured daily.

The relative neutralising antibodies were assessed in blood samples drained by heart puncture during the total exsanguination procedure in euthanasia with the use of the surrogate kit cPass SARS-CoV-2 Neutralisation Antibody Detection Kit (Cat. Number L00847, GenScript USA Inc., USA). The SARS-CoV-2 neutralising antibody detection assay adopted in our study quantifies the S protein of SARS-CoV-2 total neutralising antibodies independent of animal species. The blocking occurs (percentage) by the interaction between the receptor binding domain (RBD) of the SARS-CoV-2 spike glycoprotein and the angiotensin-converting enzyme 2 (ACE2) human cell surface receptor attached to the plate and horseradish peroxidase (HRP)-labeled RBD used for detection. Lung sample genome was extracted and purified using the RNA/DNA 300kit H96 in Janus G3 and Janus Chemagic automatic extractor (Perkin-Elmer, Waltham, USA). SARS-CoV-2 genome amplification of the lungs was realised with a molecular kit for E region (Bio-Manguinhos, Rio de Janeiro, Brazil). The extraction, purification and amplification of SARS-CoV-2 genome amplification were realised following the manufacturer’s instructions.

*Histopathology and SARS-CoV-2 Ag detection in respiratory tract* - Tissue samples from the oropharyngeal tract and lungs were collected during necropsies and were frozen and stored at -70ºC until further analysis. A portion of each sample was stored in 10% buffered formalin (pH 7.0) and embedded in paraffin according to standard methods. Paraffin blocks were sectioned at 4 μm and stained with hematoxylin-eosin. Slides were examined under brightfield microscopy. Additionally, the immunofluorescence staining for SARS-CoV-2 nucleocapsid protein (Thermo Fisher, USA) and pan-cytokeratin (Biocare Medical, USA) was realised. All immunofluorescence assays were counterstained with Evans Blue and 4’,6-Diamidino-2-Phenylindole (DAPI). The tissue sections were analysed with Zeiss LSM 710 Confocal Microscope.

*Ethics* - Our experimental research protocol was previously approved by the Ethics Committee in the Use of laboratory animals of Fiocruz (Protocol number: CEUA LW-9/20).

## RESULTS

**Fig. 2:**
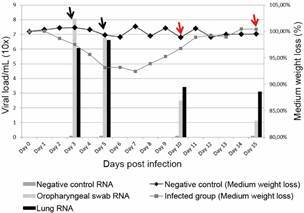
clinical and virological findings of A.2 severe acute respiratory syndrome coronavirus 2 (SARS-CoV-2) infection in Golden Syrian hamsters. The columns indicate the viral load of A.2 SARS-CoV-2 RNA found in the oropharynx and lung and the lines indicate the medium weight loss (%) of Golden Syrian hamster infected with A.2 SARS-CoV-2 strain. Arrows in black represent seronegative plasma samples at 3 and 5 days post-inoculation (DPI), and arrows in red represent seropositive plasma samples at days 10 DPI and 15 DPI, indicating when hamsters infected with A.2 SARS-CoV-2 strain presented SARS-CoV-2 specific neutralising antibodies.

*Clinical and virological findings* - The infected group with the A.2 strain did not show any clinical manifestation of SARS-CoV-2 infection and lost about 5%-8% of their body weight at 3-7 DPI. After 10 DPI, all animals recovered their body weight from the pre-inoculation step. The most elevated viral RNA load was detected in the oropharynx (10^8^ RNA copies) and lung (10^6.6^ RNA copies) at 3 DPI and 5 DPI respectively. An important reduction of respiratory viral RNA content occurred from 10 DPI onwards, when neutralising antibodies were detectable in animals (Fig. 2).

**Fig. 3:**
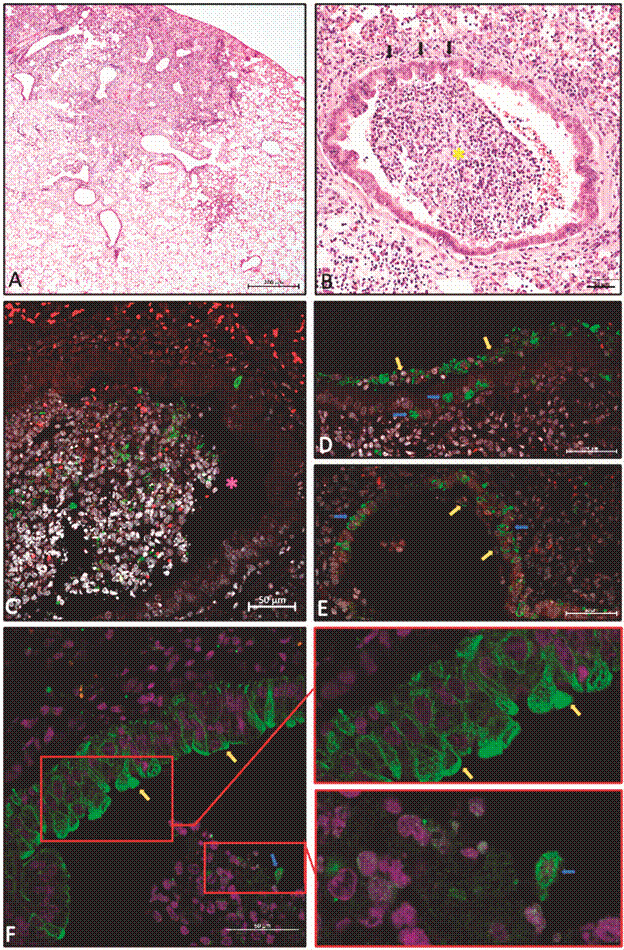
presence of respiratory tract epithelial cell plugs and concomitant occurrence of segmental lesions in the lungs of hamsters infected with severe acute respiratory syndrome coronavirus 2 (SARS-CoV-2). (A) Low-magnification histological image showing the contrast between an intensely injured area (top right) and a normal neighbouring area (left). (B) Intensely damaged lung area, highlighting the bronchus semi-obstructed by plug cells (^*^yellow), mostly epithelial from the respiratory tract. Black arrows indicate points of contact between plug cells and bronchial wall epithelial cells, which are injured. (C) Immunofluorescence for the nucleocapsid protein of SARS-CoV-2, showing the presence of several cells infected by the virus in a cell plug trapped in a bronchial lumen (^*^pink). (D) and (E) Presence of the SARS-CoV-2 nucleocapsid protein, both in remaining plug cells (yellow arrows) and in bronchial epithelial cells where they were installed (blue arrows). (F) Immunofluorescence for pan-cytokeratin shows positive epithelial cells on the bronchial layer and inside the bronchiolar lumen with a more circular morphology, along with lymphocytes (amplified image). Paraffin-embedded lung sections: (A) and (B) HE is staining, brightfield microscopy (Metasystems slide scanner), 20x objective lens/ 0.8 NA; (C), (D) and (E) immunofluorescence for SARS-CoV-2 nucleocapsid protein (green), counterstained with Evans Blue (red blood cells and cytoplasm of some cells in red) and DAPI (nuclei in white). (F) Immunofluorescence for mouse monoclonal pan-cytokeratin (Biocare Medical, cat. CM043-C) in green, counterstained with Evans Blue (red blood cells and cytoplasm of some cells in red) and DAPI (nuclei in white). Confocal laser scanning microscopy (Zeiss LSM 710), objective lens 40x/ 1.3 NA.4.

*Respiratory tract histopathological findings and SARS-CoV-2 Ag detection* - Histopathological analysis of the lungs revealed severe segmentary pneumonitis with remarkable sub-epithelial lymphoeosinophilic infiltrates in some bronchi, thus contributing to the detachment of epithelial cells. Of note, some of the epithelial cells were morphologically changed, suggesting a direct cytopathic viral effect on the primary site of viral replication, the nasopharynx. There were some foci of lymphocyte infiltration through vascular walls towards the intima, with subendothelial inflammatory infiltration, followed by erosion and micro haemorrhages. Histological analysis revealed a contrast between an intensely injured area and a normal neighbouring area (Fig. 3A). We also detected vascular endothelial injury with karyorrhexis and microthrombus; trapped lymphocytes into dilated lymphatic vessels around a bronchial tree and the pulmonary artery; white thrombus and severe lymphatic congestion; a high number of lymphocytes in inflammatory infiltrates contributing to arterial wall erosion and possibly further development of artery-bronchus or alveolar fistula.

Surprisingly, we observed cell plugs into some terminal bronchial lumen. These plugs were composed mainly of respiratory tract epithelial cell clusters with mucus, cell debris and lymphocytes that were probably aspirated from the upper respiratory tract. Fig. 3B shows focally extensive damage at the parenchyma with a partial bronchial obstruction by a plug of cells, mostly epithelial from the upper respiratory tract. Immunofluorescence for SARS-COV-2 nucleocapsid protein reveals heterogeneity of expression in the same plug since some highly positive epithelial cells contrast with low positive and negative ones (Fig. 3C). Additionally, immunofluorescence for SARS-CoV-2 nucleocapsid protein shows the presence of several cells infected by SARS-CoV-2 in a cell plug trapped in a bronchial lumen (Fig. 3D-E). To confirm the epithelial origin of some plug cells, cytokeratin was used in an immunofluorescence assay. The pan-cytokeratin immunofluorescence showed positive epithelial cells on the bronchial layer and inside the bronchiolar lumen along with lymphocytes (Fig. 3F).

## DISCUSSION

Rhesus monkeys are considered the best translational animal model for studying COVID-19 treatment because they express Rhesus angiotensin-converting enzyme 2 (rhACE2)^3^ with high homology with hACE2 receptor.^12^ The infection of rhesus monkeys with SARS-CoV-2 can recapitulate a moderate^13^ or severe COVID-19 disease in humans.^3^ ^,^ ^12^ ^,^ ^14^ However, the high-cost ABSL3 for non-human primates are unaffordable for low-income countries. Therefore, the Golden Syrian hamster model may represent a relevant and less expensive approach to assess the efficacy of new vaccines, therapeutic antibodies, and other antiviral products before moving to phase-I clinical trials in developing countries.

In our study, infected Golden Syrian hamsters did not show any detectable clinical signs of respiratory dysfunction and all animals survived throughout the experiment. However, from 3-5 DPI, the lungs of hamsters inoculated with SARS-CoV-2 showed morphological changes in epithelial cells. These morphological changes suggest a direct cytopathic viral effect, which is also described in oral epithelial desquamate cells of severe COVID-19 disease patients.^15^ Besides that, infected hamsters developed SARS-CoV-2-induced vascular changes, commonly associated with human infection: endothelial injury, microthrombus, lymphocytes trapped into dilated lymphatic vessels around a bronchial tree and the pulmonary artery; white thrombus and severe lymphatic congestion; the presence of lymphocyte infiltrates into arterial walls with erosion and development of alveolar fistula.^16^ Similar to our results, SARS-CoV-2 infected macaques have also shown vascular changes with micro thrombosis and micro haemorrhagic events in the alveolar space at 5 DPI.^3^ ^,^ ^17^ ^,^ ^18^

Even though the respiratory tract of infected hamsters was severely affected at the onset of SARS-CoV-2 infection, at 10 DPI all animals showed histological signs of recovery and weight gain. This recovery could have occurred because normal areas of the lungs may have assumed a mechanism of compensatory activation to prevent hypoxemia.^19^ Highly injured pulmonary areas contrasting with normal ones may be explained by the presence of infectious plugs obstructing or even occluding some terminal bronchi, thus contributing to segmentary pneumonitis despite the absence of cough reflex.

Besides that, the infectious plugs may play a role in SARS-CoV-2 wide and long-lasting dissemination into the parenchyma (*viral metastasis hypothesis*), with a continuous immune system stimulation of the respiratory tract because of the closed interaction between the SARS-CoV-2 infected epithelial cells (plugs) and the non-infected epithelial cell (also in the plug or the deep terminal bronchiole). A similar feature, with epithelial plugs followed by obstructive respiratory distress, was described in the early seventies in necropsies of children with severe lung dysfunction after infection with the respiratory syncytial virus. The infant necropsies revealed severe viral bronchiolitis and interstitial pneumonia.^20^ On the other hand, the interstitial neutrophil infiltration, fibrosis, and bronchial terminal lumen obstruction caused by granulation tissue, frequently described in viral acute bronchiolitis^21^ were not found in our A.2 SARS-CoV-2 infected hamster model. Alternatively, plugs can be ingested during a strong cough episode or through mucociliary clearance. The ingestion of these infectious plugs may contribute to the SARS-CoV-2 dissemination through the digestive tract, with recurrent immune stimulation and inflammation. This “trojan horse” phenomenon has been described in the literature for neutrophils and dendritic cells in other viral infections.^22^ In conclusion, our findings showed that hamsters infected with A.2 SARS-CoV-2 exhibit infectious epithelial plug spreading, bronchial and vascular damage, including the fistulae, and cell lymphatic trapping and infiltration. These changes in their respiratory tract could be one of the reasons for human long-lasting COVID-19 disease.
